# Effects of periodontal clinical database software in resident training during COVID-19 pandemic: a prospective observational study

**DOI:** 10.1186/s12909-022-03289-8

**Published:** 2022-04-01

**Authors:** Wenjun Zhu, Rongmei Feng, Yun Fu

**Affiliations:** Department of Periodontology, Guanghua School of Stomatology, Hospital of Stomatology, Sun Yat-Sen University, Guangzhou, China

**Keywords:** COVID-19, Dental education, Dental residency, Periodontitis

## Abstract

**Background:**

Dental residents in Guangdong, China, had fewer medical practice opportunities because of the pandemic of COVID-19. This study aimed to evaluate whether a case-based learning (CBL) approach using a periodontal clinical database software (PCDS) could improve residents' achievement in the exam of the standardized residency training (SRT) program.

**Methods:**

Forty-four dental residents volunteered and completed this trial. Within 12 weeks, all residents admitted periodontal patients 5 days a week and participated in a case-based learning course using PCDS once a week. Two online case-based examinations were used to evaluate their diagnostic and therapeutic performance before and after training. The total accuracy rate of examinations and the accuracy rate of subitems were analyzed using paired samples T-test. The Bonferroni correction is used for multiple testing adjustments, and *p* < 0.05 was considered statistical significance.

**Results:**

After training, the total accuracy rate of SRT exams raised from 65 to 76%. There was a significant difference in the accuracy rate before and after training (Mean = 0.103, SD = 0.141, *p* < 0.001). The accuracy of radiographic examination (type of alveolar bone absorption and hard tissue lesion of tooth) and making treatment plan was significantly improved after training (*p* < 0.005). However, residents' performance in diagnosing periodontitis and predicting the prognosis of affected teeth was not improved.

**Conclusions:**

The PCDS and CBL method effectively improved the residents' achievement in SRT examination, especially in identifying the type of resorption of alveolar bone and the hard tissue lesion of a tooth by radiographic examination and making an appropriate treatment plan for a periodontitis patient. More effective teaching approaches are needed to improve residents' accuracy of diagnosis of periodontitis using the 2018 classification in China.

## Background

Coronavirus disease 2019 (COVID-19) is a contagious disease caused by severe acute respiratory syndrome coronavirus 2 (SARS-CoV-2). Fever, cough, dyspnea, myalgia, and fatigue are the most commonly reported manifestations in COVID-19 patients [1]. In addition, a considerable number of COVID-19 patients show oral symptoms such as taste and smell alteration [2], oral ulcers [3], and gingival pain and bleeding [4]. It is reported that the angiotensin-converting enzyme 2 (ACE2) receptor, which is the target site of SARS-CoV-2, is expressed in gingival epithelial cells, taste cells of the tongue [5], and salivary glands [6]. Therefore, the oral cavity susceptible to SARS-CoV-2 is considered to provide the potential site of human-to-human viral transmission and present symptoms characterizing COVID-19 [7].

COVID-19 has affected dentists, dental academics, and dental students by changing lifestyles and causing psychological stress [8] because the virus is transmitted by contact with droplets and aerosols [9]. Periodontal treatment using ultrasonic devices became a challenge during the COVID-19 pandemic. According to Wajeeh's research [10], 83.8% of dentists chose manual scaling instead of ultrasonic scaling to minimize aerosol generation. Except for dental emergencies, all non-essential dental procedures were canceled or postponed in February 2020 in Guangzhou, China. Residents were asked to stay home until the outbreak was under control. Our data showed that the number of residents in the Department of Periodontology of Sun Yat-sen University dropped to zero in February 2020. Only a few residents participated in clinical practices until July 2020. The average medical fee per resident decreased from 17,073 yuan (from Sep 2019 to Jan 2020) to 12,855 yuan (from May 2020 to Jul 2021). These data implicated fewer patients and fewer medical practice opportunities for residents.

The didactic lectures were switched to online because dental schools have been locked down to prevent transmission [11]. Asynchronous and synchronous methods used in E-teaching during the COVID-19 pandemic were found to be effective, especially those receiving feedback [12]. On the contrary, clinical skill training, which is the essential infrastructure of dental residency education, was severely affected. It is reported that 74.7% of students considered clinics and laboratory environments more effective than E-learning in acquiring clinical skills [13]. Periodontal residents in the Standardized Residency Training (SRT) program in Guangdong, China, should finish a certain number of periodontal procedures within three months of rotation. They needed to extend clinic work hours or shorten their vacations to compensate for the lost clinical time. Simulation with a mannequin helps polish students' surgical skills in the laboratory. However, learning from actual patients is essential for medical students to improve the accuracy of diagnosis, assessment of prognosis, and the ability to make a treatment plan. In order to compensate for the reduction of practice opportunities, we developed a periodontal clinical database software (PCDS) containing both common and rare periodontal diseases in the present study. Residents could learn about different types of periodontal diseases in their spare time without face-to-face contact through this software. The case-based learning (CBL) approach has been widely used in medical education. It is a student-centered teaching methodology that exposes students to real-world scenarios that need to be solved using their reasoning skills and existing theoretical knowledge [14]. We speculated whether using clinical database software and the CBL approach together could improve periodontal residents' performance in the standardized residency training program exam.

## Methods

### Study design

The present study is a prospective observational study aimed to evaluate whether the periodontal clinical database software and case-based learning method could effectively improve the residents' achievement in the SRT examination.

**Fig. 1:**
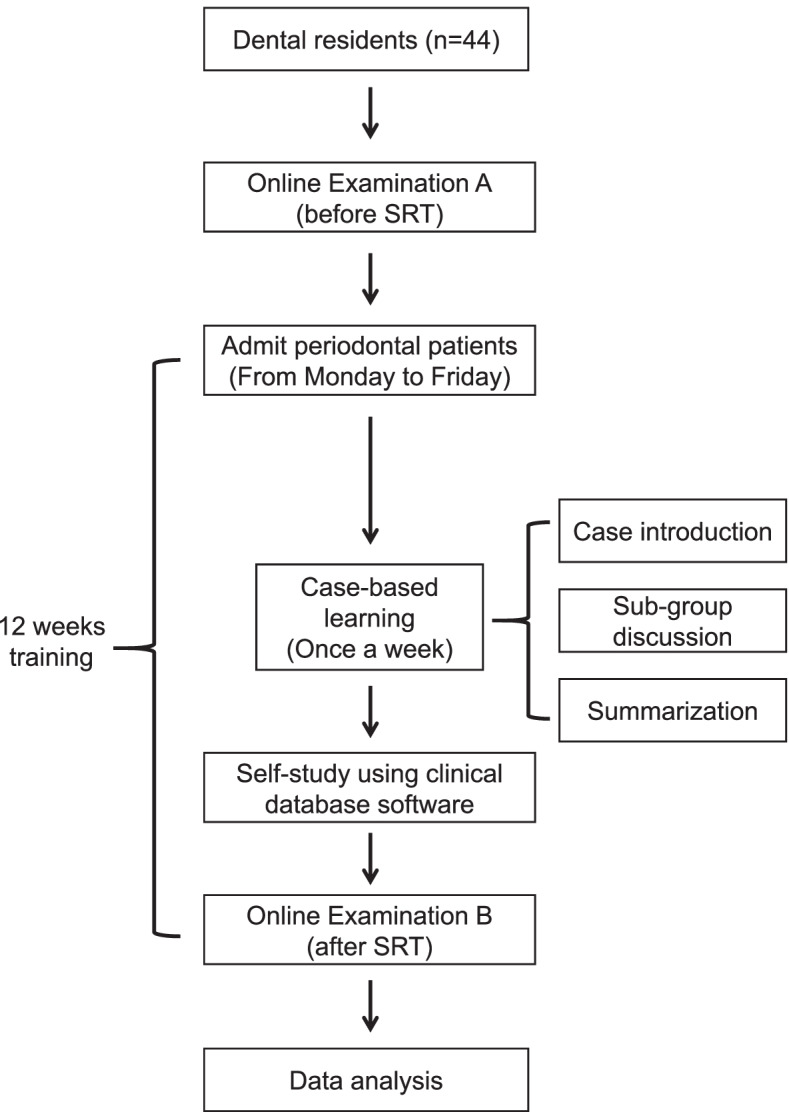
Training setting for periodontal residents

**Fig. 2:**
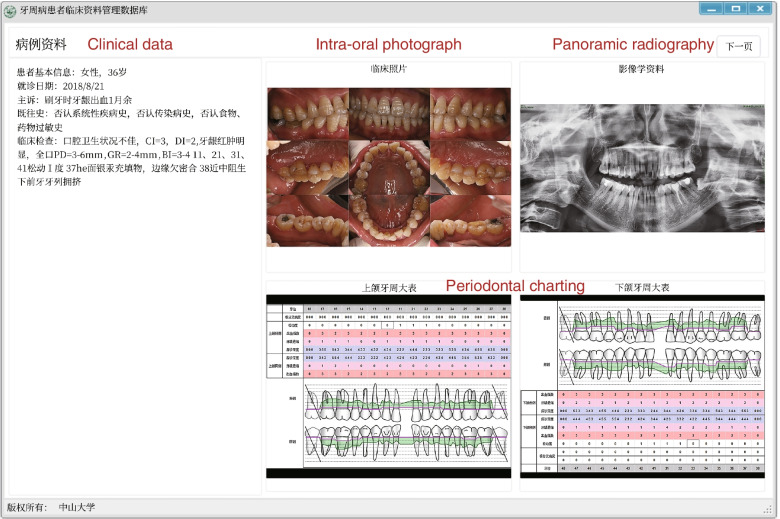
The user interface of periodontal clinical database software (first page)

**Fig. 3:**
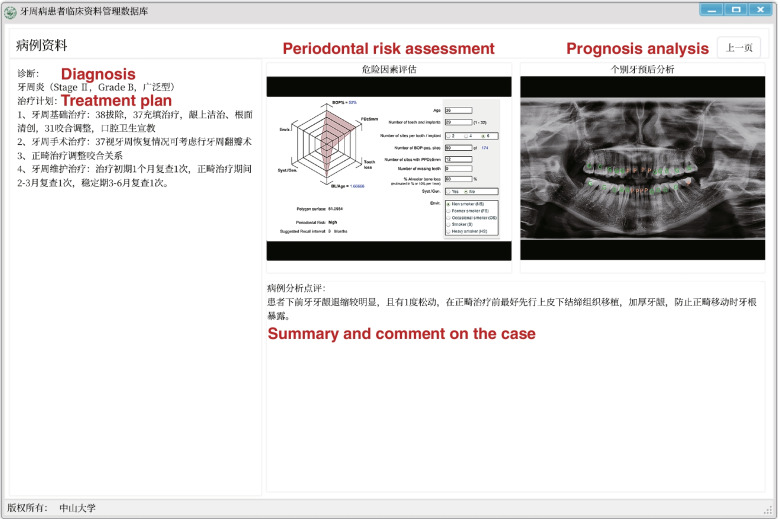
The user interface of periodontal clinical database software (second page) The second page comprises the diagnosis, treatment plan, periodontal risk assessment, prognosis analysis, and summary of the case. The photographs are blurred for privacy protection

Figure 1 shows the training setting for periodontal residents. Dental residents (from Hospital of Stomatology, Sun Yat-sen University, Guangzhou, China) volunteered and completed this training program (from April 2020 to May 2021). All residents in the present study have obtained a bachelor's degree in stomatology. All residents treated periodontal patients from Monday to Friday for 12 weeks under the supervision of clinical teachers (periodontists). In addition, students participated in a case-based learning course once a week. The data of periodontal cases, including intraoral photographs, periodontal examination chart, panoramic radiography, evaluation of periodontal prognosis, and treatment plan, were recorded in PCDS (Figs. 2 and 3), which allow all students to learn the cases at home. At the beginning of CBL classes, the teacher briefly introduced the case and raised several questions about the diagnosis, prognosis, and treatment plan. The residents discussed in a sub-group manner and reported their answers to the questions group by group. At last, the teacher analyzed the case's difficult points and summarized the case.

The residents were asked to complete the online tests before and after SRT. The training program comprises 12 weeks' daily outpatient practice and a case-based learning course once a week.

The first page comprises the clinical data of patients (basic information without a real name, chief complaint, medical history, results of intra-oral examination), intra-oral photographs, panoramic radiography, and periodontal charting of the upper and lower jaw. The photographs are blurred for privacy protection.

### Sample size estimation

**Fig. 4:**
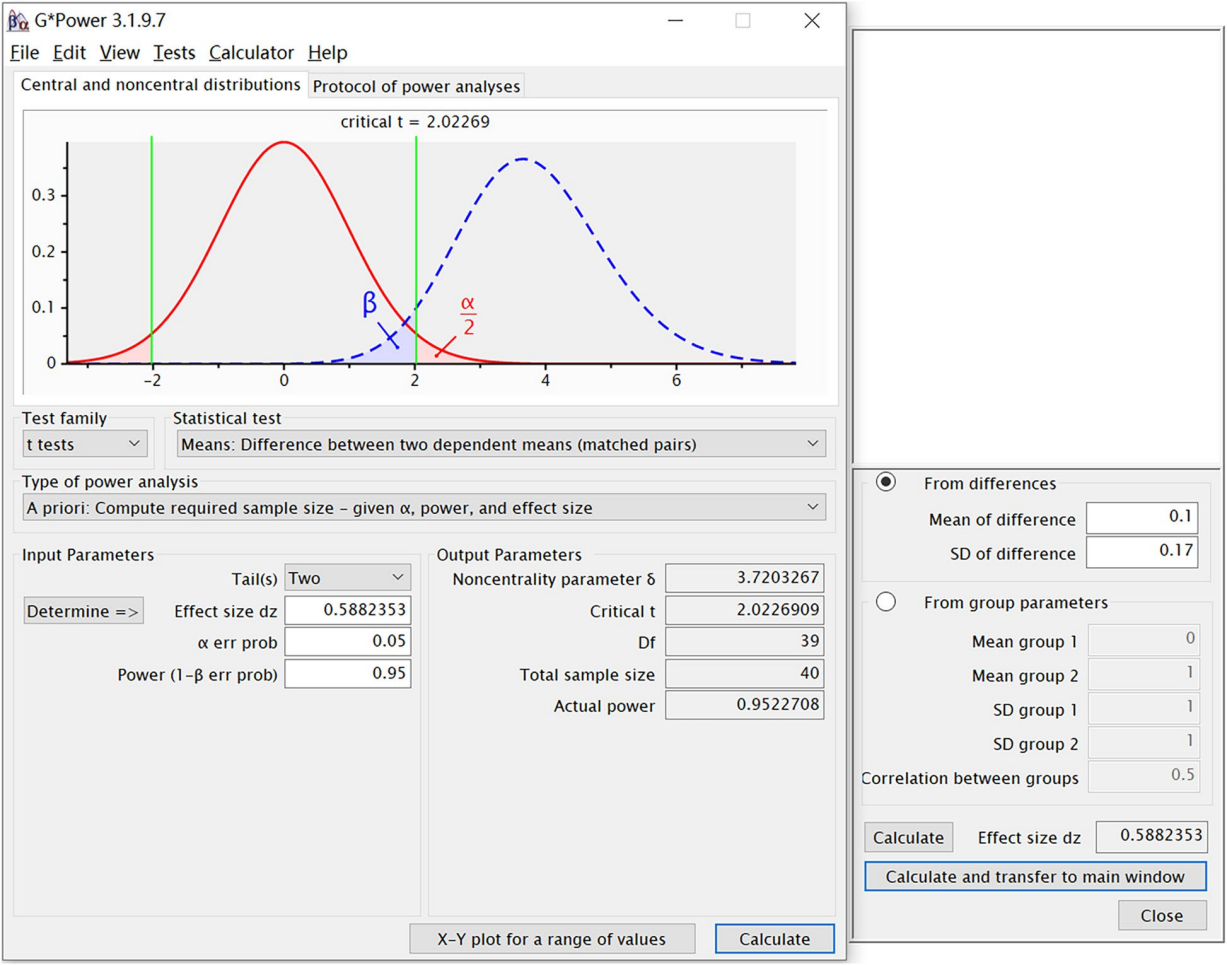
Sample size estimation using G*Power software

In the present study, the significant level α was set at 0.05, and the statistical power of test 1-β was set at 95%. According to our preliminary study, the standard deviation was 0.17, whereas the mean difference before and after training was 0.1. The sample size of this study estimated by the software G*Power [15] (version 3.1.9.7) is 40 (Fig. 4). Therefore, the sample size of the present study was determined to be more than 40.

The paired sample t-test was used in the present study. The significant level α and the statistical power of test 1-β are set at 0.05 and 0.95, respectively. The effect size is calculated by using data from the previous study (mean of difference = 0.1, the standard deviation of difference = 0.17). The estimated sample size is 40.

### Evaluation of periodontal diagnostic and treatment capabilities

All students were asked to complete the online case-based examination within 30 min before and after the training program. Each examination comprises two periodontal cases, which were used 1 to 3 years ago in the final exam of the Standardized Residency Training program of the hospital of stomatology, Sun Yat-sen University. Each case has several questions concerning radiography, diagnosis, prognosis, and treatment plan. Details of the composition of the examination are shown in Table 1.

**Table 1:** Composition of examination before and after SRT

Subitem	Number of questions in Exam A (before SRT)	Number of questions in Exam B (after SRT)
radiographic examination (Type of alveolar bone absorption)	2	2
radiographic examination (Extent of alveolar bone absorption)	2	2
radiographic examination (hard tissue lesion of a tooth)	1	1
radiographic examination (peri-apical lesion)	1	2
Diagnosis of periodontitis (Stage)	2	2
Diagnosis of periodontitis (Grade)	2	2
Diagnosis of periodontitis (Extent and distribution)	2	2
Diagnosis of other dental diseases	1	2
Prognosis of affected teeth	2	2
Treatment plan	9	6
Total	24	23

The questions aim to evaluate students' abilities in radiographic interpretation, diagnosis of periodontitis using 2018 classification, diagnosis of other dental diseases, prediction of prognosis, and making an appropriate treatment plan.

### Statistical analysis

The accuracy of examination before and after SRT was expressed as mean ± standard deviation. The total accuracy rate of examinations (primary outcome measure) and the accuracy rate of subitems (secondary outcome measures) were analyzed using paired samples T-test. Shapiro–Wilk test was conducted to verify the normal distribution of data. The Bonferroni correction was used for multiple testing adjustments. All data were analyzed by SPSS Statistics software (Version 22.0, IBM, USA), and *p* < 0.05 was considered statistical significance.

## Results

In the present study, 8 male (18.2%) and 36 female (81.8%) residents were recruited. Nine residents (20.5%) were employees from other hospitals in Guangdong province. Another 9 residents (20.5%) were employed by the Hospital of Stomatology, Sun Yat-sen University. The rest of the 26 residents (59.9%), who were unemployed, were postgraduate students from Sun Yat-sen University (Table 2).

**Table 2:** Main characteristics of 44 dental residents

Gender	Male	8 (18.2%)
	Female	36 (81.8%)
Identity	Residents employed by other hospitals	9 (20.5%)
	Residents employed by Hospital of Stomatology, Sun Yat-sen University	9 (20.5%)
	Postgraduate students from Sun Yat-sen University (unemployed)	26 (59.9%)

A total of 44 residents were recruited in the study, including 8 males (18.2%) and 36 females (81.8%). Nine residents (20.5%) were employees from other hospitals in Guangdong province, whereas another 9 residents (20.5%) were employees of the hospital of stomatology, Sun Yat-sen University. The rest of 26 residents (59.9%) were postgraduate students from Sun Yat-sen University, and all of them were unemployed.

The results of the Shapiro–Wilk test (*p* = 0.479) showed that the difference in the total accuracy between the two exams fit the normal distribution. In addition, a paired-samples t-test was conducted to compare the total accuracy of exam A and exam B (Table 3). There was a significant difference in the total accuracy rate of exams before and after training (Mean = 0.103, SD = 0.141, *p* = 0.001).

**Table 3:** Comparison of total accuracy of exams before (exam A) and after (exam B) training

Pair	Mean ± SD	*p*-value
Exam B—Exam A	0.103 ± 0.141	0.001**

A paired-samples t-test was conducted to compare the total accuracy of exam B with exam A. ** indicates *p* < 0.01

**Fig. 5:**
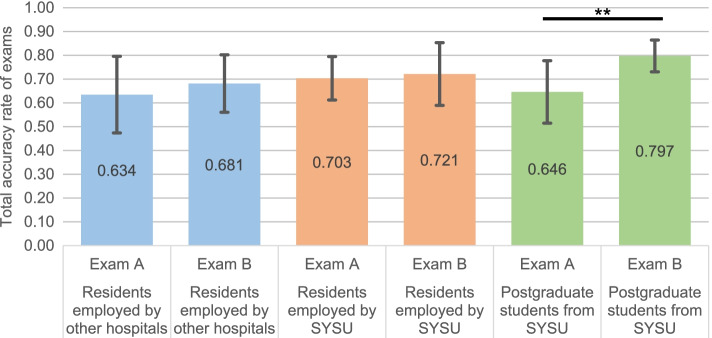
Total accuracy of exam A and exam B clustered by resident identity Total accuracy of SRT exam at baseline (exam A) and after training (exam B) was expressed as mean ± SD according to the resident identity. Error bar represented standard deviation. ** indicates *p* < 0.01

The effects of gender and resident identity on the accuracy of exams were evaluated via independent samples t-tests. The accuracy rate before and after training was not affected by gender (0.094 ± 0.147 vs. 0.140 ± 0.110, *p* = 0.413) (Table 4). In contrast, the improvement after training was mainly contributed by postgraduate students (0.152 ± 0.141, *p* = 0.001). Residents from Sun Yat-sen University (0.016 ± 0.136, *p* = 0.731) or other local hospitals (0.047 ± 0.124, *p* = 0.289) showed a slight improvement in the exam after training, though the difference is not statistically significant (Table 5 and Fig. 5).

**Table 4:** Effects of gender on total accuracy between exam A and exam B

Gender	Exam B—Exam A (Mean ± SD)	*p*-value
Female	0.094 ± 0.147	0.413
Male	0.140 ± 0.110	

An independent samples t-test was used to assess whether gender affected the total accuracy of exams before and after training

**Table 5:** Effects of resident identity on total accuracy between exam A and exam B

Identity	Exam B—Exam A (Mean ± SD)	*p*-value
Residents employed by other hospitals	0.047 ± 0.124	0.289
Residents employed by Hospital of Stomatology, Sun Yat-sen University	0.016 ± 0.136	0.731
Postgraduate students from Sun Yat-sen University (unemployed)	0.152 ± 0.141	0.001**

Paired-samples t-tests were conducted to assess whether resident identity affected the total accuracy of exams before and after training. **Statistically significant (Bonferroni correction. *p* < 0.016)

Furthermore, the accuracy rate of subitems in two exams was also analyzed by paired-samples t-test (Bonferroni correction). Results (Table 6) showed that the accuracy of radiographic examination (identification of the type of alveolar bone absorption and hard tissue lesion) and making a treatment plan increased significantly after training. However, residents' performance in the diagnosis of periodontitis and predicting the prognosis of affected teeth were not improved.

**Table 6:** Comparison of subitem accuracy between exam A and exam B using paired-samples t-test

Subitem	Exam B—Exam A (Mean ± SD)	*p*-value
radiographic examination (Type of alveolar bone absorption)	0.307 ± 0.593	0.001**
radiographic examination (Extent of alveolar bone absorption)	0.102 ± 0.351	0.060
radiographic examination (hard tissue lesion of tooth)	0.659 ± 0.479	0.001**
radiographic examination (peri-apical lesion)	0.102 ± 0.351	0.060
Diagnosis of periodontitis (Stage)	-0.239 ± 0.451	0.001**
Diagnosis of periodontitis (Grade)	-0.125 ± 0.507	0.109
Diagnosis of periodontitis (Extent and distribution)	0.057 ± 0.309	0.229
Diagnosis of other dental diseases	-0.011 ± 0.476	0.875
Prognosis of affected teeth	-0.159 ± 0.370	0.007
Treatment plan	0.246 ± 0.235	0.001**

^**^Statistically significant (Bonferroni correction. *p* < 0.005)

**Fig. 6:**
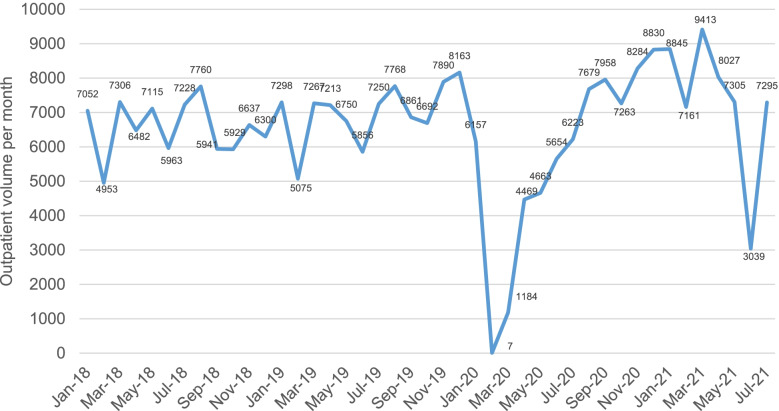
The outpatient volume of the Department of Periodontology, Sun Yat-sen University

As shown in Fig. 6, the outpatient volume of the Department of Periodontology dropped drastically in February 2020 and March 2020. With the control of the pandemic in China, the hospital outpatient volume gradually returned to the level before the outbreak. However, it was still affected by sporadic local cases and local health policy. For example, only the patients with a negative nucleic acid test result against SARS-CoV-2 within 72 h can be admitted because of the community-transmitted cases in Guangzhou in July 2021.

The outpatient volume was about 6000 to 8000 per month before the outbreak of COVID-2019. It decreased drastically in February 2020 and slowly recovered not until August 2020. Nevertheless, it was still affected by sporadic local cases and local health policy.

**Fig. 7:**
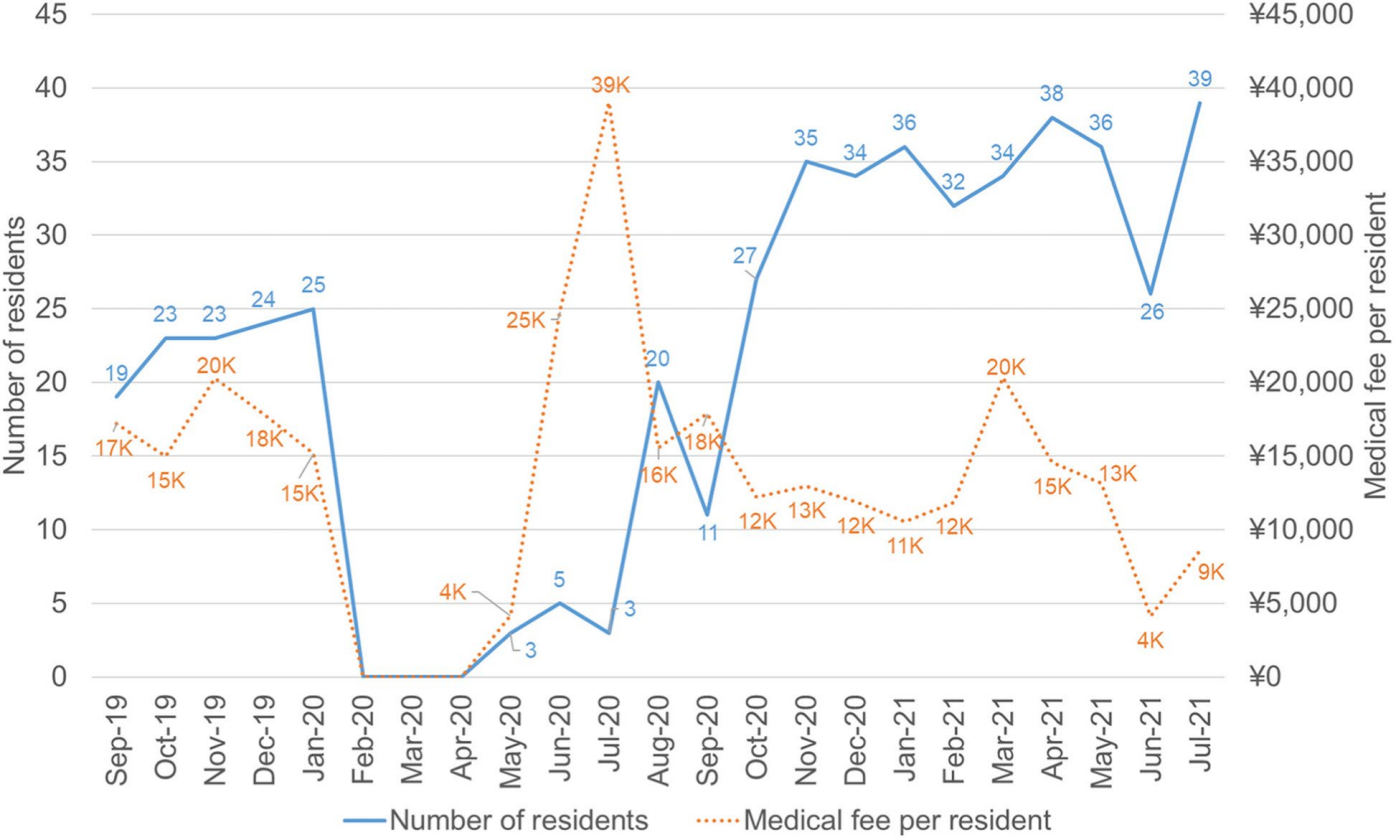
The number and the medical fee of residents

Figure 7 showed that the number of residents dropped to zero in February 2020, and only a few residents returned and participated in clinical practices until July 2020. The average medical fee per resident decreased from 17,073 yuan (from Sep 2019 to Jan 2020) to 12,855 yuan (from May 2020 to Jul 2021). These data implicated fewer patients and fewer medical practice opportunities for residents.

The blue line (left coordinate axis) indicated the number of residents in the Department of Periodontology. It dropped to zero in Feburay 2020, recovered to the average level in October 2020, and got higher than the average level after November 2020. The orange dot line (right coordinate axis) indicated the average medical fee per resident (unit in Chinese yuan). It went down after November 2020 since more and more residents got back in the clinic.

## Discussion

Coronavirus disease 2019 (COVID-19) is a contagious disease caused by severe acute respiratory syndrome coronavirus 2 (SARS-CoV-2). COVID-19 is mainly transmitted via the airborne route by inhaling droplets or aerosols that infected people breathe out [16]. Dental treatment, including ultrasonic scaling, air polishing, and prophylaxis procedures, produces contamination (splatter, droplets, and aerosol) in the presence of suction. Studies [17] found that aerosol contamination can spread up to 3 m and extend to the inside of personal protective equipment such as masks and face shields. Therefore, only urgent dental diseases were attended to during the early stage of the COVID-19 outbreak in the Hospital of Stomatology, Sun Yat-sen University (Feb 2020). The biggest challenge in resident training has been to postpone direct patient care, a vital component of the dental curriculum. According to Abbasi et al.'s research, only a few (11.4%) students were confident to practice on patients after E-learning, suggesting that clinical training is indispensable in medical education [13].

For now, there is no consensus on what should be included in the periodontal case record. Teachers and students usually use Microsoft Powerpoint software to record their periodontal cases one by one, making it inconvenient and inefficient to organize or share cases with others. The periodontal clinical database software used in the present study stipulates the standard format of the data, including intra-oral photography, periodontal charting, panoramic radiography, diagnosis, and treatment plan. One can easily find qualified cases according to the restricted conditions (for example, smokers or pregnant women cases) or share with others. Moreover, the PCDS also contained a summary and comments of cases that helped residents better understand the difficulty and comprehend how the treatment plan was made. In addition, residents have insufficient knowledge and limited training in rare diseases [18]. For instance, hereditary gingival fibrosis is a rare periodontal disease that causes gingival enlargement of the entire mouth [19], whose incidence rate is 1 in 750,000. There were only 4 cases from 2012 to 2020 in the Department of Periodontology, Sun Yat-sen University, which means residents will hardly come into one case in their 3-month training rotation. Nevertheless, with the help of the PCDS, students have more chances to learn from these rare cases.

The goal of CBL is to prepare students for clinical practice by using authentic clinical cases [20]. In CBL, both the students and teachers prepare in advance, and there is guidance to the discussion so that important learning points are covered. However, the results indicated that only postgraduate residents from SYSU benefited from the training, whereas residents employed by SYSU or other hospitals gained slight improvement. One possible explanation is that the postgraduate residents paid more attention to the training and showed more interest in learning. Koole et al. [21] reported that dental students who actively engaged in discussions during case-based learning had better learning outcomes than those who passively observed the ongoing discussions. Further experiments focusing on student engagement are needed to verify this hypothesis.

In the present study, residents' performance was improved by 10% in the total accuracy after training. Most improvements were seen in radiographic examination (determining the type of resorption of alveolar bone and the hard tissue lesion of a tooth) and making an appropriate treatment plan for a patient. However, they failed to increase the accuracy of the diagnosis of periodontitis using 2018 new classification criteria. The new classification of periodontal diseases was brought up in 2018 [22]. It recommended characterizing periodontitis using a staging and grading system that describes the clinical presentation and other elements that affect clinical management, prognosis, and potentially broader influences on both oral and systemic health. However, the old 1999 classification system is still the main content in the newest edition of the Periodontology textbook published in 2020 [23], in which the new classification is only briefly introduced. Residents in this study started to learn the 2018 classification when they were recruited.

Abou-Arraj et al. [24] reported that periodontal postgraduates showed a greater level of agreement in the diagnosis of periodontitis compared to all dental students group (second-year and four-year dental students), suggesting daily practice can refine the learning of making an accurate diagnosis. Interestingly, they also found that while the overall agreement on the diagnosis was suboptimal, predoctoral and postgraduate students seemed to agree on treatment planning (nonsurgical periodontal therapies) irrespective of their level of training. Similar results were found in the present study. Residents made better treatment planning even though they could not diagnose periodontitis accurately. One possible explanation is that most periodontitis patients share a similar treatment strategy. Residents in this study were also found to overestimate disease severity after training (percentage of overdiagnosis before and after training was 64.5% and 91.4%, respectively). A more effective teaching method is needed to improve the accuracy of diagnosis of periodontitis.

The limitations of this study were that residents were recruited from only one dental hospital, and the evaluation of residents' performance was carried out immediately after the training through an online examination. Therefore, the results of the current study should be interpreted with caution. Further research, with a long-term follow-up, larger sample sizes, and residents from other universities is needed to ascertain the beneficial effects mentioned above. We also recommend establishing criteria for clinical data in dentistry that facilitate communication and sharing. Video and other forms of media such as virtual reality and augmented reality can also be used in presenting clinical cases or surgical procedures, which may help enhance student engagement in class [25].

## Conclusions

The periodontal clinical database software and case-based learning method effectively improved the residents' achievement in SRT examination, especially in determining the type of resorption of alveolar bone and the hard tissue lesion of a tooth by radiographic examination and making an appropriate treatment plan for a periodontitis patient. More effective teaching approaches, especially when the outpatient volume is low, are needed to further improve residents' accuracy of diagnosis of periodontitis using the 2018 classification in China.

## Data Availability

The datasets used and/or analyzed during the current study are available from the corresponding author on reasonable request.

## Abbreviations

ACE2: Angiotensin-converting enzyme 2
CBL: Case-based learning
COVID-19: Coronavirus disease 2019
PCDS: Periodontal clinical database software
SARS-CoV-2: Severe acute respiratory syndrome coronavirus 2
SRT: Standardized residency training
